# Investigation of PDE5 effect on NOS in nasal polyp pathophysiology

**DOI:** 10.1007/s00405-025-09362-4

**Published:** 2025-04-05

**Authors:** Vahit Mutlu, Zülküf Kaya, Zekai Halıcı, Ayşegül Tavacı Özçelik, Abdullah Serdar Topatan

**Affiliations:** 1 Department of Otorhinolaryngology, Atatürk University Faculty of Medicine, Yakutiye, Erzurum 25240 Turkey; 2 Department of Pharmacology, Atatürk University, Yakutiye, Erzurum Turkey

**Keywords:** Nasal polyp, NOS, Pathophysiology, PDE5

## Abstract

**Purpose:**

Nasal polyps are masses resulting from chronic mucosal inflammation. Nitric oxide (NO) has recently attracted attention in nasal polyps as it plays an important role in both acute and chronic inflammation. One of the important mechanisms controlling NO production is phosphodiesterase (PDE) enzymes. The enzyme phosphodiesterase type 5 (PDE5) is an important regulator of cyclic guanosine 3‘-5’-monophosphate (cGMP) signalling. PDE5 inhibitors increase intracellular cGMP concentration by inhibiting cGMP degradation and prolong NO signalling. NO is thought to cause nasal congestion because it increases microvascular permeability and causes mucosal oedema. The aim of our study was to investigate the role of PDE5, inducible nitric oxide synthase (iNOS), and endothelial nitric oxide synthase (eNOS) in the pathophysiology of nasal polyps with mucosal oedema in histopathology.

**Methods:**

Nasal mucosal tissues were obtained from 25 patients with nasal polyps who underwent endoscopic sinus surgery as the study group and 25 patients who underwent rhinoplasty as the control group. eNOS, iNOS and PDE5 levels were measured in nasal mucosal tissues.

**Results:**

The mean age was 47.40 ± 16.33 years in the nasal polyp group and 35.44 ± 12.47 years in the normal group, and 64.0% (*n* = 16) of both groups were male. ELISA measurements showed that PDE5 levels were significantly decreased and iNOS and eNOS levels were significantly increased in the nasal polyp group compared with the control group.

**Conclusıon:**

This study suggest that iNOS, eNOS, and PDE5 may play important roles in the pathophysiology of nasal polyps.

## Introduction

Nasal polyps are translucent, oedematous, pale grey masses resulting from chronic mucosal inflammation in the nasal and paranasal cavities [1]. Nasal polyps originating from the middle meatus and ethmoid region are usually bilateral, mostly benign lesions [2]. It affects approximately 1–4% of the general population. The most common complaint of patients is nasal congestion. Nasal congestion can cause headaches, anosmia, hyponasal speech, decreased taste and smell, snoring, rhinorrhea, and nasal discharge [3]. Nasal irrigation, topical and systemic corticosteroids, antibiotics, and endoscopic sinus surgery are all options for the treatment of nasal polyps. Recurrence of polyps and symptoms is very common even after pharmacological and surgical treatment [4]. In a study, it was present that nasal polyps recurred in 40% of patients 6 months after surgery [5]. At present, the mechanism of polyp formation is still not fully understood [6]. For this reason, the treatment process of nasal polyps is quite problematic. Due to the high recurrence rate after surgical treatment and the adverse effects of long-term use of systemic corticosteroids, deeper investigation of nasal polyp pathophysiology and the development of pharmacotherapeutic approaches are necessary [7, 8].

In recent studies on nasal polyps, a chronic inflammatory disease, nitric oxide (NO) has attracted attention because it plays an important role in both acute and chronic inflammation. NO, which is found at high levels in the nasal cavity, especially in the paranasal sinuses, has been identified as an important mediator in the pathogenesis of various respiratory diseases [9]. NO has many functions in host defence, ciliary activity, inflammation, and regulation of vascular tone in the respiratory tract [2]. NO is synthesised from L-arginine by the nitric oxide synthase (NOS) enzyme family. Three different isoforms of this enzyme have been identified. The constitutive isoforms of endothelial nitric oxide synthase (eNOS) and neuronal nitric oxide synthase (nNOS) are expressed in endothelial and neuronal tissues, respectively. Its inducible isoform, inducible nitric oxide synthase (iNOS), is expressed in various cells in the setting of inflammation [10]. Structural NOS, eNOS, and nNOS release small amounts of NO under physiological conditions, whereas inducible NOS, iNOS, can catalyse the formation of large amounts of NO. The main source of NO in the upper respiratory tract is iNOS, which is triggered by type 2 inflammation and eosinophilia [11]. NO has no specific receptor on the cell membrane and increases the intracellular concentration of cyclic guanosine 3‘-5’-monophosphate (cGMP) in vascular smooth muscle cells by crossing the plasma membrane and activating soluble guanylate cyclase (sGC). Increased cGMP decreases intracellular Ca2 + concentration and leads to relaxation of vascular smooth muscle cells. Since NO causes microvascular leakage and stimulation of glandular secretions by vasodilation, it is thought to play a role in mucosal oedema, which is the main feature of nasal polyps [12]. cGMP increased by NO is rapidly inactivated by phosphodiesterases (PDE). The intracellular concentration of cGMP is determined by the balance between GC and PDE activities induced by NO [13].

PDEs disrupt the phosphodiester bond of cyclic adenosine monophosphate (cAMP) and cGMP, converting them to their inactive forms, respectively [14]. In particular, phosphodiesterase-5 (PDE5) converts cGMP to the inactive 5’-GMP form. PDE5 inhibitors increase intracellular cGMP by inhibiting cGMP hydrolysis. Increased cGMP causes prolongation of NO signalling in vascular smooth muscle cells, leading to smooth muscle relaxation and vasodilation [15]. PDE5 inhibitors, which cause vasodilation in the corpus cavernosum, are used in the treatment of erectile dysfunction because they promote erection [16]. It is well known that the most common side effects of PDE5 inhibitors are a feeling of nasal congestion and stuffy or runny nose [17]. The NO signalling prolonged by PDE5 inhibition is thought to increase microvascular permeability, leading to oedema and nasal congestion [12]. In the light of these data, we think that one of the causes of mucosal oedema in nasal polyps is increased NO as a result of the pathophysiological disturbance in PDE5 synthesis and release. The aim of our study was to show the role of PDE5 in the pathophysiology of nasal polyps by looking at the levels of PDE5 and iNOS, eNOS in nasal polyp tissue.

## Material methods

Fifty patients who applied to Atatürk University Faculty of Medicine, Otorhinolaryngology Outpatient Clinic between 2023 and 2024 participated in our study. All participants were included in the study after giving written informed consent. Twenty-five of these patients were diagnosed with nasal polyps and underwent endoscopic sinus surgery, and the other 25 were control group patients who underwent rhinoplasty due to anatomical variations without any nasal mucosal pathology. Patients who participated in our study did not receive systemic or topical intranasal steroid and antibiotic treatment for four weeks before surgery. In addition, patients with cystic fibrosis, immunodeficiency, Samter’s triad, Churg-Strauss syndrome, granulomatous polyangiitis, primary ciliary dyskinesia, unilateral NP (anthracoanal polyp), fungal sinusitis (EPOS 2020), individuals under the age of 18 or over the age of 78, pregnant and lactating women were excluded from our study.

In our study, nasal polyp tissues were obtained from the middle turbinate of 25 patients who underwent endoscopic sinus surgery and from the inferior turbinate of 25 patients who underwent rhinoplasty. The tissues were stored at -80 C. PDE5 and iNOS, eNOS levels were measured by ELISA method.

### ELISA

The tissues included in our study were stored at -80 °C until the time of biochemical analysis. The samples were then subjected to physical homogenisation in liquid nitrogen using a TissueLyser II (QIAGEN). 100 mg was weighed for each sample. Weighed tissues were homogenised in 1 mL PBS homogenate buffer in an Eppendorf tube using the TissueLyser II device. Then centrifugation was performed in the ELISA kit as recommended by the manufacturer. After the supernatants were obtained, PDE5 and iNOS, eNOS levels were measured. Protein amounts were measured using the Lowry method and levels were normalised to cell protein concentrations.

## Results

The demographic characteristics of the 25 nasal polyp and 25 normal participants included in the study are presented in Table 1. Accordingly, 64.0% (*n* = 16) of both groups were male. The mean age was 47.40 ± 16.33 years in the nasal polyp group and 35.44 ± 12.47 years in the normal group, and a significant difference was found between the groups in terms of mean age (*p* = 0.005). However, no significant difference was found between the two groups in terms of gender, mean weight, height, and body mass index (BMI) (*p* > 0.05).

**Table 1:** The demographic characteristics of the participants included in the study

			Nasal Polyp Group	Normal Group	*P*
Gender	Male	N	16	16	1.0*
		%	64.0%	64.0%	
	Female	N	9	9	
		%	36.0%	36.0%	
Age	Mean		47.40	35.44	**0.005**†
	95% Confidence Interval for Mean	Lower Bound	40.66	30.30	
		Upper Bound	54.14	40.58	
	Median		43.00	30.00	
	Std. Deviation		16.33	12.47	
	Minimum		22	20	
	Maximum		78	67	
	Interquartile Range		26	19	
Weight	Mean		75.92	77.52	0.640†
	95% Confidence Interval for Mean	Lower Bound	71.23	72.31	
		Upper Bound	80.61	82.73	
	Median		76.00	79.00	
	Std. Deviation		11.354	12.623	
	Minimum		49	57	
	Maximum		103	110	
	Interquartile Range		13	16	
Height cm	Mean		172.16	173.16	0.700†
	95% Confidence Interval for Mean	Lower Bound	168.28	169.51	
		Upper Bound	176.04	176.81	
	Median		172.00	172.00	
	Std. Deviation		9.393	8.844	
	Minimum		150	156	
	Maximum		190	188	
	Interquartile Range		13	13	
BMI	Mean		25.5861	25.8269	0.799†
	95% Confidence Interval for Mean	Lower Bound	24.3205	24.3609	
		Upper Bound	26.8516	27.2930	
	Median		25.3086	26.3128	
	Std. Deviation		3.06589	3.55164	
	Minimum		19.14	18.52	
	Maximum		31.10	34.72	
	Interquartile Range		4.25	3.78	

* Chi-square test

† Student-t test

When the participants were compared according to their medical history, only the history of allergic rhinitis showed a difference between the groups; 64.0% of the nasal polyp group had allergic rhinitis, while 28.0% of the normal group had allergic rhinitis and there was a statistically significant difference (*p* = 0.011). No significant difference was found between the groups in terms of aspirin hypersensitivity, asthma, atopy history, smoking and alcohol use (*p* > 0.05) (Table 2).

**Table 2:** The comparison of the groups according to the medical history

			Nasal Polyp Group	Normal Group	*P*
Aspirin Hypersensitivity	Yok	N	24	25	1.0*
		%	96.0%	100.0%	
	Var	N	1	0	
		%	4.0%	0.0%	
Asthma	Yok	N	18	19	0.747†
		%	72.0%	76.0%	
	Var	N	7	6	
		%	28.0%	24.0%	
Atopy history	Yok	N	13	19	0.077†
		%	52.0%	76.0%	
	Var	N	12	6	
		%	48.0%	24.0%	
Smoking	-	N	18	22	0.157†
		%	72.0%	88.0%	
	+	N	7	3	
		%	28.0%	12.0%	
Alcohol use	-	N	23	23	1.0*
		%	92.0%	92.0%	
	+	N	2	2	
		%	8.0%	8.0%	
Allergic Rhinitis	-	N	9	18	**0.011**†
		%	36.0%	72.0%	
	+	N	16	7	
		%	64.0%	28.0%	

* Fisher’s Exact test

† Pearson Chi-square test

### Biochemical analysis results

**Fig. 1:**
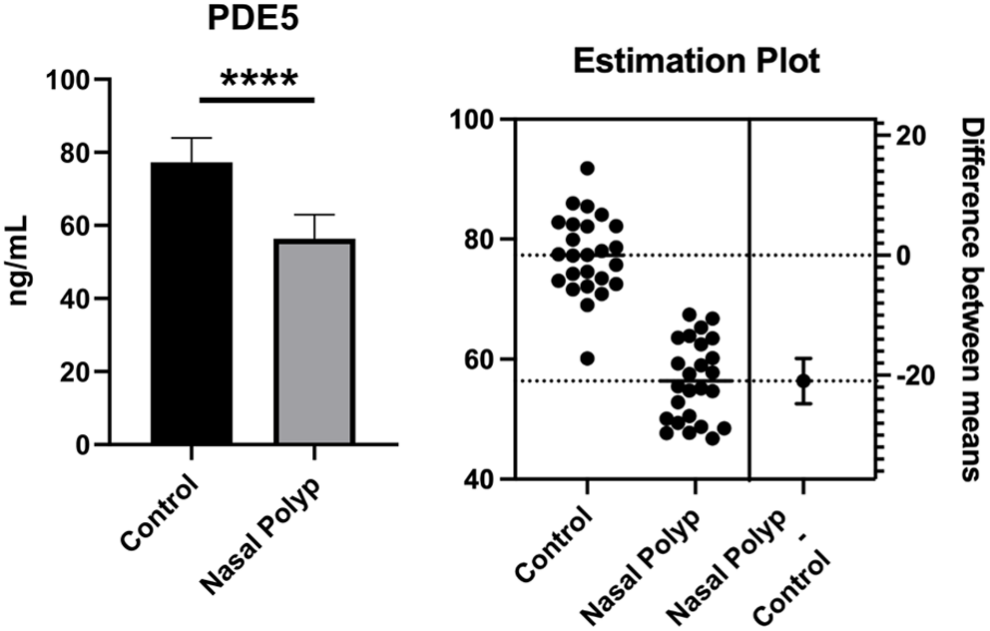
PDE5 levels obtained from nasal polyps and healthy nasal mucosa tissues. **** Significant difference at *P* < 0.001 compared to healthy group

As shown in Fig. 1, it was observed that the PDE5 level in the nasal polyp group was significantly decreased compared to the healthy group when measured by the ELISA method (*p* < 0.001).

**Fig. 2:**
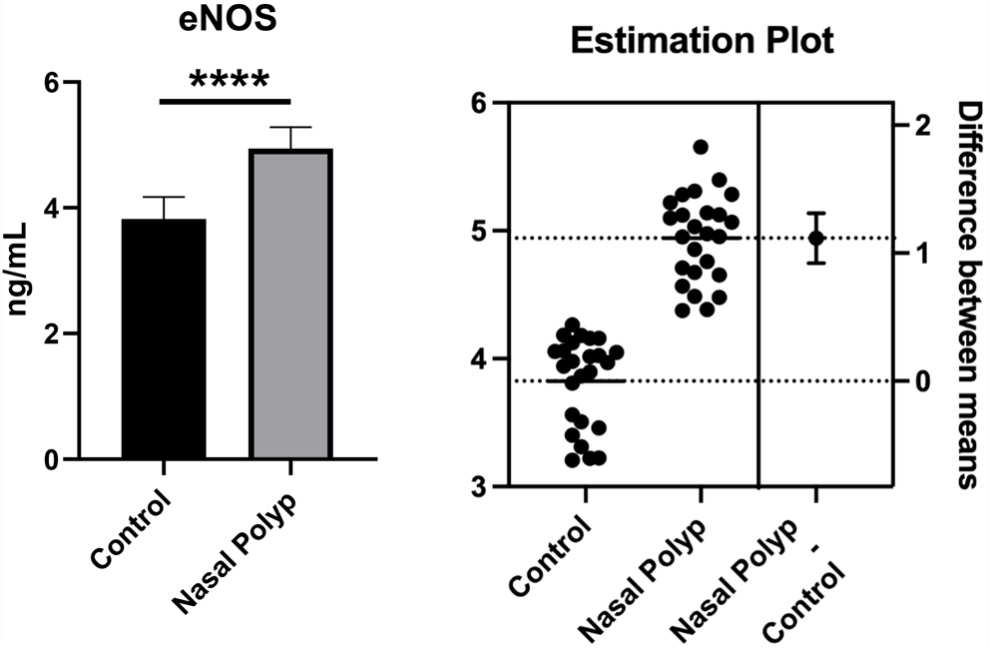
eNOS levels obtained from nasal polyps and healthy nasal mucosa tissues. **** Significant difference at *P* < 0.001 compared to healthy group

As shown in Fig. 2, it was observed that the eNOS level in the nasal polyp group was significantly increased compared to the healthy group when measured by the ELISA method (*p* < 0.001).

**Fig. 3:**
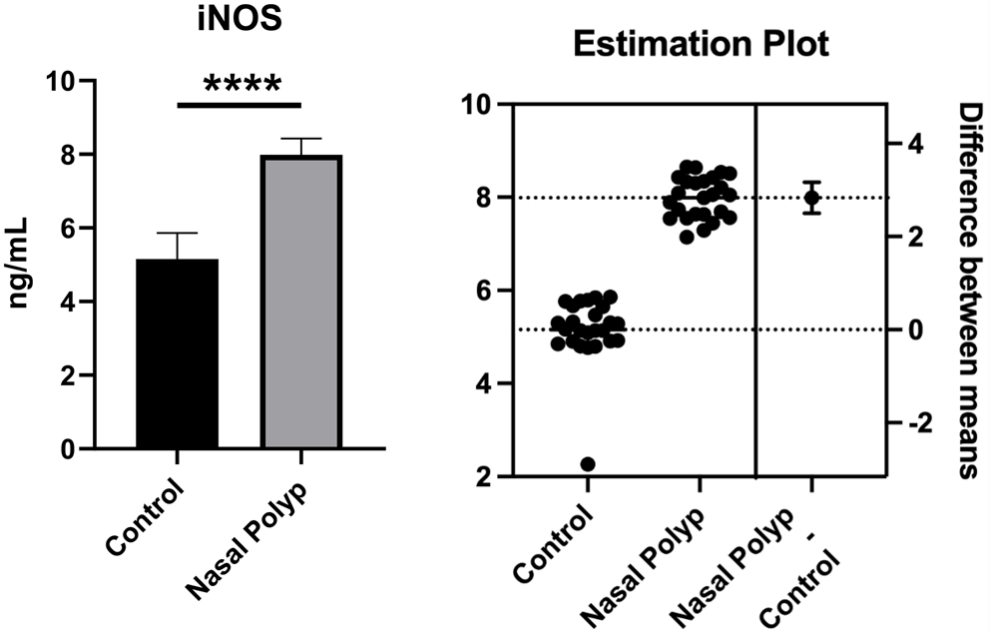
iNOS levels obtained from nasal polyps and healthy nasal mucosa tissues. **** Significant difference at *P* < 0.001 compared to healthy group

As shown in Fig. 3, it was observed that the iNOS level in the nasal polyp group was significantly increased compared to the healthy group when measured by the ELISA method (*p* < 0.001).

### Statistical analysis

The data obtained from the study were analysed using SPSS V27 software (IBM, USA). Numerical variables were presented as mean and standard deviation, and categorical data were presented as percentages and frequencies. The normal distribution of the numerical data was determined by looking at the skewness and kurtosis values, which were found to be between − 1.96 and + 1.96, indicating that the data had a normal distribution. The Student t-test was used for the statistical analysis of two independent groups of numerical variables, and the chi-squared test was used for the analysis of two categorical variables. *P* < 0.05 was used for statistical significance.

All biochemical data were analysed using GraphPad Prism. Measured data are presented as mean ± standard deviation. Quantitative data were analysed using an unpaired Student’s t-test to compare the two groups.

## Discussion

In our study, PDE5, eNOS and iNOS levels were measured by ELISA in tissues obtained from patients with nasal polyps who underwent endoscopic sinus surgery and rhinoplasty for anatomical variations.We found that PDE5 levels decreased statistically significantly in the nasal polyp group compared with the control group, and eNOS and iNOS levels increased in parallel with decreased PDE5 levels.

There is a growing belief that NO may be a biomarker for various inflammatory pathologies in the upper and lower airways [18]. NO is an important signalling molecule involved in many physiological and pathological processes, including regulation of blood flow, platelet function, neurotransmission, immunity and inflammation [19, 20]. NO is synthesised from the amino acid L-arginine by NOS enzymes [21]. There are three isoforms of the NOS enzyme. Of these, eNOS and nNOS are calcium-dependent and constitutive isoforms that produce NO at lower levels, whereas iNOS is a calcium-independent inducible isoform that produces NO at higher levels [22]. NO promotes the conversion of guanosine triphosphate (GTP) to the second messenger cGMP by activating sGC [13]. Increased cGMP as a result of conversion leads to increased calcium sequestration and mediates vascular smooth muscle relaxation [21]. NO, which is found at high levels in the nasal cavity, particularly in the paranasal sinuses, increases airway resistance by causing changes in the diameter of the nasal cavity, and contributes to oedema formation by causing blood extravasation [9]. NO is also thought to play a role in the formation of mucosal oedema in nasal polyps [12]. NO has a half-life of a few seconds and therefore many studies have examined the presence of eNOS, iNOS to assess NO expression [11, 23, 24]. In particular, iNOS has been shown to be highly expressed in allergic or inflammatory conditions [25]. In a study conducted in patients with chronic rhinitis, a chronic inflammatory disease, a significant positive correlation was found between the degree of inflammation and the expression of iNOS [26]. A study in patients with allergic rhinitis also showed a significant difference in iNOS expression compared to controls [27]. Studies of nasal polyps, which develop as a result of chronic inflammation, have shown that both eNOS and iNOS activity are increased compared to normal nasal mucosa [10, 12, 28, 29]. Increased cytokines in nasal polyps increase iNOS expression and thus NO production [30]. NO produced by iNOS and eNOS activity and increased cGMP have been shown to modulate mucosal inflammation, chronic sinusitis and associated polyp formation [12]. In our study, eNOS and iNOS levels were increased in nasal polyp tissue compared to normal nasal mucosa. However, excluding the PDE enzyme when evaluating the effects of NO and its second messenger cGMP would lead to an incomplete evaluation. Because one of the most important mechanisms controlling NO production is the PDE enzymes. Intracellular cGMP increased by NO is rapidly inactivated by PDEs [13]. When we looked at the literature, there were studies that investigated the relationship between nasal polyps and NOS. However, there are no studies investigating the relationship between PDE and nasal polyps, which are closely related to NO.

PDEs are a family of enzymes that regulate cellular levels of second messengers such as cAMP and cGMP [31]. Eleven different PDE families have been identified to date [32]. Each PDE family has unique properties, and each modulates different regulatory pathways in the cell. The enzyme PDE5 is widely expressed in airway smooth muscle cells and vascular smooth muscle cells [33]. PDE5 specifically hydrolyses cGMP. PDE5 inhibitors selectively inhibit cGMP hydrolysis and increase intracellular cGMP [34]. Increased cGMP due to PDE5 inhibition causes prolongation of NO signalling in vascular smooth muscle cells, leading to smooth muscle relaxation and vasodilation [15, 35]. Increased levels of NO and cGMP, and consequently increased activity in the nasal tissue, contribute to the development of nasal polyposis in the nasal tissue. In our study, we showed that while the PDE5 levels decreased, the amount of eNOS and iNOS increased in nasal polyp tissue. While eNOS and iNOS are increased in nasal polyp tissue, the presence of PDE5 inhibition at the same time causes a prolongation of the duration of action of NO. This suggests that PDE5, in addition to iNOS and eNOS, may play an important role in the physiopathology of nasal polyp development. Another data point supporting our study and our findings is that one of the most common side effects of PDE5 inhibitors is nasal congestion [17, 36]. Studies have shown that sildenafil, a PDE5 inhibitor, reduces nasal patency and nasal airflow [9, 11]. It is thought that PDE5 inhibition may cause nasal congestion due to vasodilation caused by prolongation of NO signalling and changes in nasal cavity diameter. Sildenafil, a PDE5 inhibitor, has also been shown to increase iNOS, and eNOS levels [37–39].

Limitations of our study include the different mean ages and rates of allergic rhinitis between the control and study groups, the single-centre design and the small sample size. It needs to be supported by larger and multi-centre studies.

In conclusion, our study showed that prolonged NO signalling by PDE5 inhibition may play a role in the pathophysiology of nasal polyps. However, more detailed experimental and clinical studies on this topic are needed.
